# A new species of the rare chrysidid subfamily Loboscelidiinae from China: the third species of *Rhadinoscelidia* Kimsey, 1988 (Hymenoptera, Chrysididae)

**DOI:** 10.3897/zookeys.87.1295

**Published:** 2011-03-24

**Authors:** Jing-xian Liu, Jie-min Yao, Zai-fu Xu

**Affiliations:** College of Nature Resources and Environment, South China Agricultural University, Guangzhou, 510640, the People’s Republic of China

**Keywords:** Aculeata, Chrysidoidea, Oriental Region

## Abstract

*Rhadinoscelidia delta* Liu, Yao & Xu, **sp. n.** (Chrysididae, Loboscelidiinae) is described and illustrated based on two female specimens from Hainan province. It represents the first record of the genus *Rhadinoscelidia* Kimsey, 1988 for China. A key to the world species of this genus is given. All specimens are preserved in the Hymenopteran Collection, South China Agricultural University (SCAU).

## Introduction

Loboscelidiinae is a small subfamily of Chrysididae distributed in the Oriental and Australian regions (Cameron 1910, Kieffer 1916, Fouts 1925, Maa and Yoshimoto 1961, Lin 1964, Day 1979, Terayama et al. 1998), which includes two genera: *Loboscelidia* Westood, 1874 and *Rhadinoscelidia* Kimsey, 1988. Thirty-five species in total are recognized in Loboscelidiinae (Kimsey and Bohart 1990, Kojima and Ubaidillah 2003, Xu et al. 2006), of which thirty-three species belong to the genus *Loboscelidia*, and two species belong to the genus *Rhadinoscelidia*: *Rhadinoscelidia malaysiae* Kimsey, 1988 from Malaysia and *Rhadinoscelidia halimunensis* Ubaidillah, 2003 from Indonesia (Kimsey 1988, Kojima and Ubaidillah 2003).

Hosts of most species of this subfamily are unknown, but a few species are suggested to be parasitoids of Formicidae or Phasmatidae. Fouts (1922) suggested that Loboscelidiinae were probably myrmecophilous because they have the habitus of ants and the woolly appearance of the neck which is the characteristic of many myrmecophiles. Some species of *Loboscelidia* were inferred to be egg parasitoids of Phasmatidae (Hadlington and Hoschke 1959, Heather 1965, Riek 1970, Krombein 1983, Kimsey and Bohart 1990). However, there are no any host records of the genus *Rhadinoscelidia*. Most chrysidids are diurnal, but species of Loboscelidiinae are probably active during dusk or nocturnally based on their dark brown body and large ocelli (Kimsey and Bohart 1990).

Males of Loboscelidiinae are much more commonly collected than females (Kimsey and Bohart 1990). Recently, two specimens of the genus *Rhadinoscelidia* were discovered in Hainan, China, representing a new species, *Rhadinoscelidia delta* Liu, Yao & Xu, sp. n. It represents the first record of this genus for China.

## Materials and methods

Two specimens of the genus *Rhadinoscelidia* were collected in 2007 from Hainan. Type specimens are deposited in the Hymenopteran Collection of South China Agricultural University (SCAU).

The antenna, wings and legs on one side of the type specimen were cut off and mounted on a slide using Canada balsam. Specimens were examined and described using stereomicroscopes Leica MZ12.5 and Olympus SZ61. All pictures were made by Zeiss Imager A1 attached to a digital camera, CoolSNAP, and software Image-Pro Plus.

Abbreviations used in the descriptions as follows: POL= posterior ocellar line, the shortest distance between the posterior ocelli; MOD= mid ocellar diameter; OL= distance between middle and posterior ocelli; OOL=oculo-ocellar line, the shortest distance between the posterior ocellus and compound eye.

Morphological terminology and wing vein nomenclature are mostly based on that of Kimsey and Bohart (1990). However, *mesoscutum* and *metasoma* are used, respectively, for ‘scutum’ and ‘abdomen’.

## Taxonomy

### Rhadinoscelidia

Kimsey, 1988

Rhadinoscelidia Kimsey 1988: 77. Type species: *Rhadinoscelidia malaysiae*Kimsey 1988, original designation.

#### Diagnosis.

**Figures 1–6.**
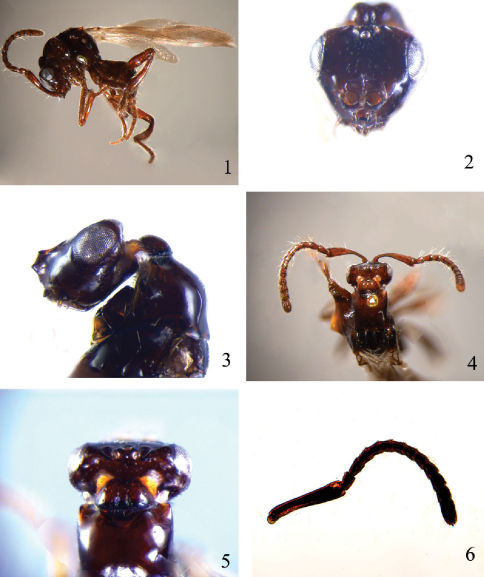
*Rhadinoscelidia delta* Liu, Yao & Xu, sp. n. holotype. **1** lateral habitus **2** head in frontal view **3** head and pronotum in lateral view **4–5** dorsal view of head and pronotum **6** antenna.

**Figures 7–12.**
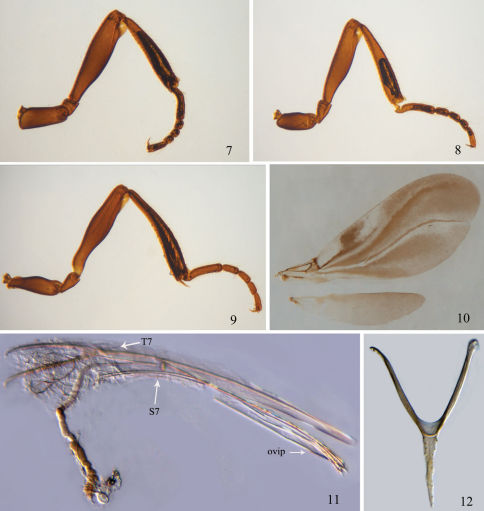
*Rhadinoscelidia delta* Liu, Yao & Xu, sp. n. holotype. **7** fore leg **8** mid leg **9** hind leg **10** fore wing and hind wing **11** seventh tergite, seventh sternite and ovipositor (indicated by T7, S7 and ovip) **12** sixth tergite.

Antenna with scape distinctly longer than head (Figs 1, 4); vertex sharply declivitous behind ocelli (Figs 3, 5); cervical projection of head with posterior shield-like expansion clearly separate from rest of head (Fig. 3); fore wing (Fig. 10) venation highly reduced, restricted to basal sixth or less; all tibiae (Figs 7–9) without longitudinal semitransparent flanges.

#### Host.

Unknown.

#### Distribution.

China (Hainan), Malaysia, Indonesia.

#### Rhadinoscelidia delta

Liu, Yao & Xu sp. n.

urn:lsid:zoobank.org:act:7A454BDD-8BDA-4E64-9992-E8FBD5CDFDBC

Figs 1 12 

##### Diagnosis (Female).

This new species can be distinguished from *Rhadinoscelidia halimunensis* Ubaidillah, 2003 by having the scape (Figs 4, 6) without transparent flange (the latter species with transparent flange), and frons (Fig. 2) with median carina not forked at upper end (the latter with median carina of frons forked at upper end); it is also easily separated from *Rhadinoscelidia malaysiae* by fore wing venation restricted to basal 1/7 (Fig. 10) (the latter species with fore wing venation restricted to basal 1/13) and first anal vein distinct (the latter with first anal vein indistinct).

##### Description.

Holotype Female. Body length 2.3 mm; fore wing length 2.5 mm. Body shiny, with sparse setae.

*Head*. Head in anterior view triangular, as wide as width of mesosoma at tegulae and 1.5 times the interocular distance; head in lateral view oblong (Fig. 3). Eyes situated on dorsal upper half of head (Fig.3). Clypeus with short and erect setae. Frontal projection (Figs 2–3) in frontal view bilobate and trapezoid, in lateral view weakly up curved and obliquely truncate, lower lateral corner with transverse carinae extending to two sides of clypeus, upper lateral corner with carinae extending backward along middle of inner margin of eye then to posterior ocelli. Frons sparsely and finely punctuate, with a reverse triangular depression area. Gena polished. Ocelli oval, with some micropunctures and short wrinkles around, MOD=2, POL=3, OL=1, OOL=8. Vertex (Figs 3, 5) abruptly sloping and angulate posteriad lateral ocelli. Cervical projection (Fig. 5) strongly constricted behind ocelli and eyes, and posteriorly expanded and shield-like dorsally, centrally with a shallow longitudinal furrow. Vertex, gena and anterior lateral sides of cervical projection with ribbon-like setae. Antenna stout (Fig. 4, 6), covered with long and dense setae, about equals to the length of body. Scape 5.0 times as long as wide (Fig. 6), curved, without transparent flange. Pedicel similar to flagellar segments, 1.8 times as long as wide. Relative proportion of length to width of flagellomeres as follows: 40 : 32; 36 : 32; 36 : 32; 36 : 32; 36 : 32; 36 : 32; 36 : 34; 32 : 37; 31 : 39; 33 : 41; 67 : 40.

*Mesosoma*. Pronotum polished, with maximum width 1.4 times as long as maximum length, proximal basal width 0.8 times as long as ultimate apical width. Lateral margin of pronotum rounded (Fig. 3), not sharp; anterior lateral sides of pronotum with short, narrow row of ribbon-like setae. Mesoscutum smooth, posterolateral projections lamellate, sparsely setose. Notauli complete, mesoscutum between notauli weakly concave. Parapsides indistinct. Tegula as long as pronotum, sparsely setose, and extending to posterior margin of scutellum. Scutellum smooth. Propodeal projection obtuse, inconspicuous.

*Legs* (Figs 7–9). Slender, with sparse bristles; all femora with small apical transparent flanges 1/8 times as long as femur; all tibiae without transparent flanges.

*Wings*. Fore wing (Fig. 10) infuscate, with hyaline streaks, covered with dense pubescence; venation highly reduced, restricted to basal 1/7; first anal vein distinct, parallel to and as long as M+Cu vein, cu-a vein indistinct, short. R1 vein 0.9 times, Rs vein 2.8 times and cu-a vein 0.3 times as long as length of stigmal vein. Hind wing veinless.

*Metasoma* (Fig. 1). First tergite subtriangular, maximum length 0.5 times the maximum width; second tergite weakly trapezoid, with maximum length 0.75 times maximum width; third tergite narrow, 0.5 times as long as wide; fourth tergite weakly exposed; all tergites smooth, scattered with sparse setae. Segments and ovipositor that retracted within metasoma as illustrated (Figs 11, 12).

*Colour*. Body dark red. Antennae and legs reddish brown.

##### Male.

Unknown.

##### Materials examined.

Holotype, female, China: Hainan, Mt. Wuzhishan (18.85°N, 109.66°E), May 16–20, 2007, Li-qiong Weng, No. 200800122. Paratype: 1 female, same data as type, No. 200800160.

##### Etymology.

The specific name derives from Greek ‘delta’, meaning triangular, referring to the triangular depression on frons.

##### Remarks.

The terminal segments and ovipositor structure of these females from China are similar to those of the genus *Loboscelidia* as described by Day (1979).

### Key to species of Rhadinoscelidia of the World

Males are not known for *Rhadinoscelidia delta* and females are not known for *Rhadinoscelidia halimunensis* and *Rhadinoscelidia malaysiae*, so the following key has limited utility.

**Table:** 

1	Male. Metasoma with five visible segments	2
–	Female. Metasoma with four visible segments. Fore wing venation restricted to basal 1/7; first anal vein distinct; frons with a reversed triangular depression near antennal sockets. China (Hainan)	*Rhadinoscelidia delta* sp. n.
2	Scape with transparent flange on basal 1/4; frons with median carina forked at upper end near anterior margin of anterior ocellus. Indonesia	*Rhadinoscelidia halimunensis* Ubaidillah
–	Scape without transparent flange on basal 1/4; frons with median carina not forked at upper end near anterior margin of anterior ocellus. Malaysia	*Rhadinoscelidia malaysiae* Kimsey

## Supplementary Material

XML Treatment for Rhadinoscelidia
